# *CaRDR1*, an RNA-Dependent RNA Polymerase Plays a Positive Role in Pepper Resistance against TMV

**DOI:** 10.3389/fpls.2017.01068

**Published:** 2017-06-28

**Authors:** Lei Qin, Ning Mo, Yang Zhang, Tayeb Muhammad, Guiye Zhao, Yan Zhang, Yan Liang

**Affiliations:** State Key Laboratory of Crop Stress Biology in Arid Areas, College of Horticulture, Northwest A&F UniversityYangling, China

**Keywords:** pepper, *CaRDR1*, tobacco mosaic virus (TMV), resistance, *Nicotiana benthamiana*

## Abstract

RNA silencing functions as a major natural antiviral defense mechanism in plants. RNA-dependent RNA polymerases (RDRs) that catalyze the synthesis of double-stranded RNAs, are considered as a fundamental element in RNA silencing pathways. In *Arabidopsis thaliana, RDR1, 2* and *6* play important roles in anti-viral RNA silencing. Expression of *RDR1* can be elevated following plant treatment with defense hormones and virus infection. *RDR1* has been studied in several crop species, but not in pepper (*Capsicum annuum* L.). Here, a *RDR1* gene was isolated from *Capsicum annuum* L., designated as *CaRDR1*. The full-length cDNA of *CaRDR1* was 3,351 bp, encoding a 1,116-amino acid protein, which contains conserved regions, such as the most remarkable motif DLDGD. The transcripts of *CaRDR1* could be induced by salicylic acid (SA), abscisic acid (ABA), H_2_O_2_, and tobacco mosaic virus (TMV). Silencing of *CaRDR1* in pepper resulted in increased susceptibility to TMV as evident by severe symptom, increased of TMV-*CP* transcript, higher malondialdehyde (MDA) content and lower antioxidant enzymes activities compared with that of control plants. *CaRDR1*-overexpressing in *Nicotiana benthamiana* showed mild disease symptom and reduced TMV-*CP* transcripts than that of empty vector (EV) following TMV inoculation. The RNA silencing related genes, including *NbAGO2, NbDCL2, NbDCL3*, and *NbDCL4* elevated expression in overexpressed plants. Alternative oxidase (AOX), the terminal oxidase of the cyanide (CN)-resistant alternative respiratory pathway, catalyze oxygen-dependent oxidation of ubiquinol in plants. It has an important function in plant defense against TMV. In addition, *CaRDR1* overexpression promoted the expression of *NbAOX1a* and *NbAOX1b*. In conclusion, these results suggest that *CaRDR1* plays a positive role in TMV resistance by regulating antioxidant enzymes activities and RNA silencing-related genes expression to suppress the replication and movement of TMV.

## Introduction

Pepper is an important vegetable crop with a wide variety of uses. In 2014, pepper production was 36.1 million tons including green fruit and dried pods harvested in 3.6 million hectares all over the world (http://www.fao.org). Virus is the most seriously threatened in pepper production, results in crop losses under field conditions. In order to defend against viral infection, plants have evolved complicated mechanisms. RNA silencing acts as an important antiviral defense mechanism (Baulcombe, 2004; Ding and Lu, 2011). A whole RNA silencing comprises three procedures, initiation, maintenance, and signal amplification. The plant dicer-like (DCL) nucleases, argonaute (AGO) proteins, RNA-dependent RNA polymerases (RDRs) are the central functional components of the RNA silencing-based antiviral defense (Baulcombe, 2004). DCLs deal with cutting double-strand RNAs into 21–24 nt small RNAs (Carmell and Hannon, 2004). AGO containing RNA-induced silencing complexes (RISCs) are incorporated into these small RNAs in RNA degradation, translational inhibition, or heterochromatin formation (Bologna and Voinnet, 2014). In Arabidopsis, DCL2, DCL3, and DCL4 target viral genomes to yield virus derived small interfering RNAs (viRNAs) of 22-, 24,- and 21-nts, respectively. Antiviral immunity is conferred by DCL4-dependent, 21-nt viRNAs with DCL2 acting as a DCL4 surrogate (Blevins et al., 2006; Bouché et al., 2006; Fusaro et al., 2006; Diaz-Pendon et al., 2007; Donaire et al., 2008). 24-nt viRNAs produced by DCL3 might be related to the perception of non-cell autonomous silencing signals (Brosnan et al., 2007; Diaz-Pendon et al., 2007). Whereas, AGO1 and AGO2 proteins are specifically involved in antiviral defense by catalyzing viral RNA cleavage (Jaubert et al., 2011).

RNA-dependent RNA polymerases (RDRs) are essential for synthesis of double-stranded RNAs (dsRNAs), which eventually cleave into small RNAs, to originate a new turn of RNA silencing (Sijen et al., 2001; Wassenegger and Krczal, 2006; Qi et al., 2009; Voinnet, 2009; Wang et al., 2010). RDRs have been identified in a wide range of plants. The first plant-encoded *RDR* was isolated from tomato (*Solanum lycopersicum*), and named as *SlRDR1* (Schiebel et al., 1998). Silencing of *SlRDR1* using virus-induced gene silencing (VIGS) significantly reduces plant defense against tobacco mosaic virus (TMV) in tomato (Liao et al., 2015). In Arabidopsis, salicylic acid (SA) treatment and viral infection can induce a *RDR* homolog. *AtRDR1* knockout mutants accumulate higher levels of viral RNAs than those of wild-type (WT) plants after infection with tobamovirus (Yu et al., 2003). The promoter of *AtRDR1* has an extensive scope response to diverse stresses and is sensitive to ABA and SA. Analysis of promoter activity has revealed that *AtRDR1* is primarily expressed in the vascular tissue system, specifically in phloem cell layers of roots (Xu et al., 2013). However, it showed TMV inoculation-induced transient upregulation of AtRDR1 expression was attributed to wounding-induced injury, but not a direct consequence of infection (Hunter et al., 2013). Similar to tomato, *NtRDRP1* was isolated from tobacco, and *NtRDRP1* transcript could be induced by viral infection or SA treatment. The transgenic *NtRDRP1* antisense transgenic plants accumulate more virus RNA and develop symptoms that both in inoculated leaves and upper uninoculated leaves (Xie et al., 2001). A *RDR1* homolog, *NbRdRP1m*, isolated from *N. benthamiana*, contains a 72-nt insert in the 5′ position of the ORF (Yang et al., 2004). *NbRdRP1m* could also be induced by SA treatment and TMV infection. *N. benthamiana* plants overexpression with a SA-induced *RDR1* gene from *Medicago truncatula* exhibit resistance to TMV, turnip vein-clearing virus and sunn hemp mosaic virus (Yang et al., 2004). Moreover, tobacco plants overexpression with *NtRDR1* from tobacco show hyper susceptibility to plum pox poty virus and other viruses, alike to *RDR6*-silenced plants (Ying et al., 2010). In potato (*Solanum tuberosum*), SA induces expression of *StRDR1*, however, knockdown of *StRDR1* gene does not increase susceptibility to the viruses, such as TMV, PVX, and PVY (Hunter et al., 2016). *OsRDR1* in rice (*Oryza sativa*) is required for *Bromovirus*–mediated RNA silencing (Chen et al., 2010). In addition, maize *ZmRdRP1* can be induced by exogenous SA, methyl jasmonate (MeJA) treatment and sugarcane mosaic virus infection (He et al., 2010). Similarly, SA and fungal (*Rhizoctonia solani* Kuhn) infection induce *GhRDR1* in cotton (*Gossypium hirsutum*; Gao et al., 2009). These results energetically put forward an important role of *RDR1* in plant antiviral defense, nonetheless, the functions of *RDR1* still remain disputed in different species.

Despite the discrepancy in published results, it is well-recognized that plant *RDR1* is involved in viRNAs biogenesis and viRNAs-mediated antiviral defense (Qi et al., 2009; Qu, 2010). However, the role of *RDR1* in pepper virus defense still remains unknown. In the present study, a *RDR1* orthologous gene in pepper designated as *CaRDR1*, was identified, and its spatial expression patterns were characterized. *CaRDR1* was predominantly expressed in pepper stem, where played a major role in nutrient transport and virus spread. Its expression was upregulated by exogenous SA, ABA, H_2_O_2_, and TMV infection. Silencing of *CaRDR1* increased the transcript level of TMV-*CP* and reduced the resistance of pepper to TMV. Overexpression of *CaRDR1* in *N. benthamiana* resulted in less accumulation of TMV-*CP* transcripts following TMV infection. In addition, the expression levels of RNA silencing related genes, such as *NbAGO2, NbDCL2, NbDCL3*, and *NbDCL4* were upregulated by TMV inoculation in *CaRDR1* expressing *N. benthamiana*. These results strongly suggest that *CaRDR1* plays a positive role in TMV resistance in pepper.

## Materials and methods

### Plant material growth conditions and treatment

Pepper (*Capsicum annuum* L.) lines P79 and P54 used in the present study were pepper inbred lines from our lab. They showed different response to TMV-U1 (Figures S1A,B, Presentation S1). The pepper seedlings were grown in a plant growth chamber under a 16/8 h light/dark period at 25/20°C. Eight-week-old seedlings of pepper used for relative expression of *CaRDR1* in root, stem and leaf. Meanwhile, *N. benthamiana* were grown under a 16/8 h light/dark period at 25/20°C. Eight -week-old seedlings were used for TMV inoculation.

For hormone treatments, leaves of 8-week-old seedlings of pepper were sprayed with 2 mM SA, 100 μM methyl jasmonate (MeJA), 100 μM ABA and 10 mM H_2_O_2_ until surface run-off (Hunter et al., 2013; Cai et al., 2015). SA and H_2_O_2_ were dissolved in distilled water, MeJA and ABA were first dissolved in absolute ethanol to prepare a 100 mM stock solution, then diluted with sterile water to a final concentration of 100 μM. The control plants were sprayed with corresponding solvent.

TMV-U1 strain was provided by Guizhou Tobacco Science Research Institute (Shi and Guo, 2012; Ge et al., 2015). TMV-U1 was mechanically inoculated on two or three lower leaves of 8-week-old seedlings by rubbing the virus (0.01 M sodium phosphate buffer, pH 7.0) with carborundum (Kim et al., 2005). Mock inoculation was performed with the buffer only. Accumulation of TMV was confirmed by RT-PCR. The primers used for the experiments were listed in Table S1.

### Cloning of CaRDR1

Total RNA was extracted from pepper leaves using Omega plant RNA kit, and cDNA was synthesized using M-MuLV reverse transcriptase (Thermo Scientific, USA). The cDNA samples were amplified by PCR: 95°C for 5 min, 35 cycles of 95°C for 30 s, 53°C for 30 s, and 72°C for 3.5 min, and then 72°C for 10 min. The primers were listed in Table S1.

### Sequence alignment and phylogenetic analysis

Through BLAST analysis in NCBI (https://blast.ncbi.nlm.nih.gov/Blast.cgi) using the sequence of CaRDR1 protein, the related RDR1 proteins amino acid sequence in various species were gained. The multiple sequence alignments of CaRDR1 and related RDR1 proteins were performed using ClustalW in the MEGA5 software package, and the boxes were drawn using the BoxShade web site (http://www.ch.embnet.org/software/BOX_form.html). The phylogenetic tree was constructed using the Neighbor-Joining (NJ) method with Poisson model and 1000 bootstrap replicates test through MEGA5 software (Saitou and Nei, 1987; Tamura, 2011). The sequence information of the proteins used for phylogenetic tree construction was listed in Table S2.

### Quantitative real-time PCR (qRT-PCR)

Total RNA was extracted using Omega plant RNA kit, and cDNA was synthesized using PrimeScript RT reagent Kit (Takara, Dalian, China). Quantitative real-time RT-PCR (qRT-PCR) was performed using SYBR® Premix Ex TaqTM from TaKaRa (China) on iQ5 Real-Time PCR Detection System (BIO-RAD Corp., Hercules, California, USA). Pepper ubiquitin-conjugating protein (*Ubi-3*) was used as the reference gene (Wan et al., 2011). Three biological replicates were performed for qRT-PCR assay. Relative expression levels of genes were determined using the comparative threshold method (2^−ΔΔCt^; Livak and Schmittgen, 2001). The primers for qRT-PCR were listed in Table S1.

### Virus induced gene silencing (VIGS)

Vectors for VIGS had previously been described (Liu et al., 2002a). For pTRV2:*CaRDR1*, a 383-bp cDNA fragment of *CaRDR1* gene was PCR-amplified using primers shown in Table S1. The fully expanded cotyledons of pepper plants were co-infiltrated with *Agrobacterium tumefaciens* strain GV3101 carrying each TRV derivative (Li et al., 2014; Jing et al., 2016). The plants were maintained at 18–22°C in a plant growth chamber with a 16/8 h light/dark period. The efficacy of gene silence was confirmed by RT-PCR.

### Vector construction and generation of transgenic *N. benthamiana*

The pepper *CaRDR1* gene was inserted into the binary vector 35S:PBI121. Then recombinant plasmid *CaRDR1*-PBI121 and plasmid PBI121 were introduced into *Agrobacterium tumefaciens* strain GV3101. *N. benthamiana* was transformed via Agrobacterium-mediated leaf transformation according the protocols of Horsch et al. (1985) and Hou et al. (2015). The plants obtained were PCR-confirmed to select positive transgenic lines (T_0_), and the seeds from T_0_ were collected for future research.

### Determination of the malondialdehyde (MDA) content and antioxidant enzyme activity

The content of MDA was measured using Thiobarbituric acid-reactive substances (TBARS) concentration (War et al., 2012). The extraction of antioxidant enzymes were executed as described by Liao et al. (2012). Leaves (0.5 g) were blended in 10 mL of 25 mM phosphate buffer (pH 7.8) with 0.2 mM EDTA and 2% (w/v) PVP. The homogenate was centrifuged at 12,000 g for 20 min at 4°C. The supernatant was collected for the enzyme activity. Superoxide dismutase (SOD) activity was measured by inhibiting the photochemical reduction of nitroblue tetrazolium (Stewart and Bewley, 1980). Catalase (CAT) activity was monitored the decrease of the absorbance at 240 nm and the activity of peroxidase was assayed using guaiacol by monitoring the absorbance at 470 nm (Madhusudhan et al., 2009).

### Statistical analysis

SAS software is used for statistical analysis. The values are represented as the mean ± standard errors of three independent experiments. Significant differences of the data were by univariate ANOVA analysis with the least significant difference (LSD) at *P* < 0.05.

## Results

### Cloning and characterization of *CaRDR1* in pepper

**Figure 1:**
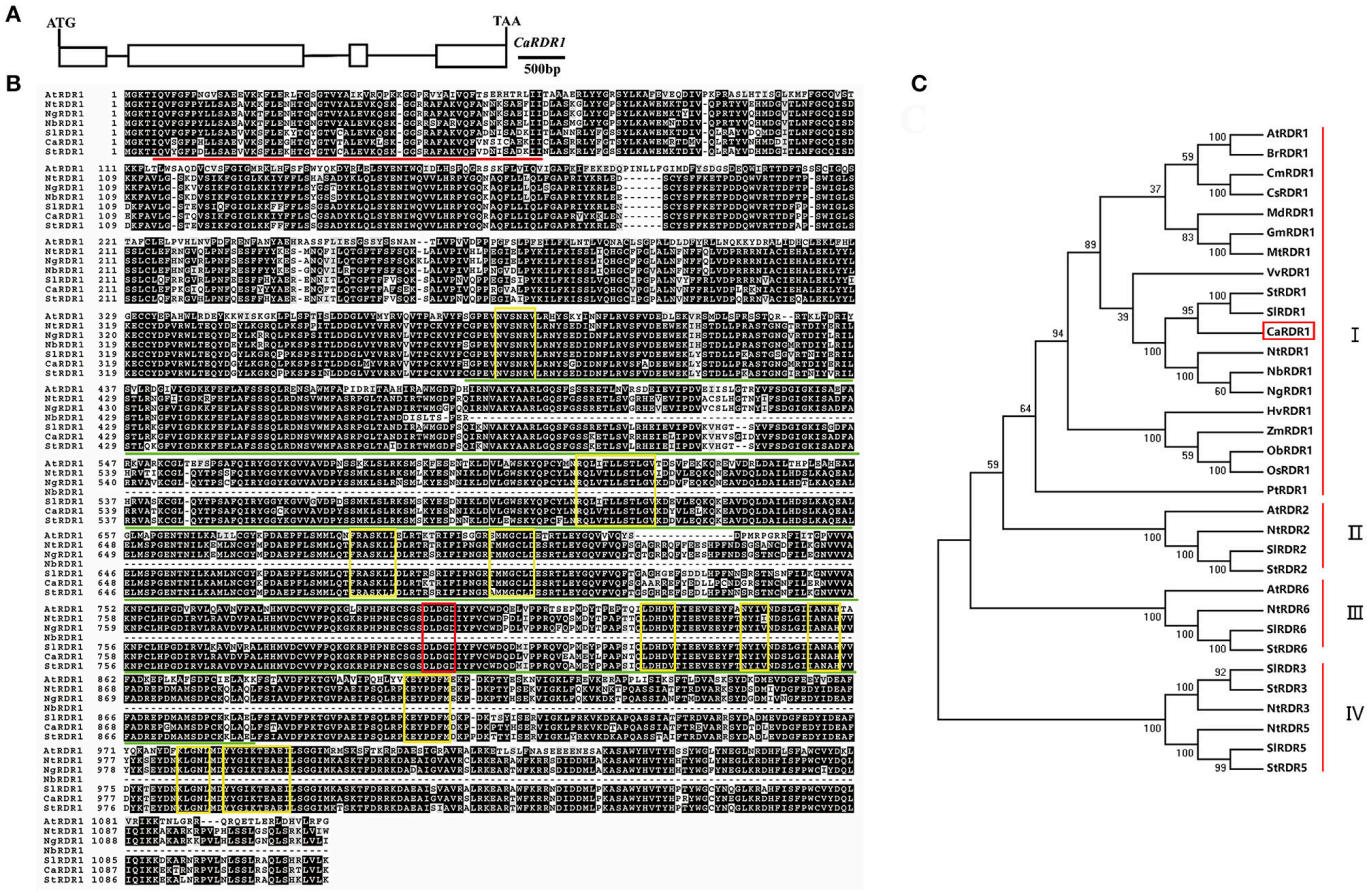
Sequence alignment and phylogenetic analyses of CaRDR1 and its homologs in various species. **(A)** Structural analysis of *CaRDR1* in pepper. Exons and introns were shown in box and line, respectively. Ca, *Capsicum annuum* L. **(B)** Sequence alignment of the amino acid of CaRDR1 with other RDR1 proteins. The identical and similar residues were shown in black and gray, respectively. The highly conserved regions for RDR1s were highlighted in yellow box, and signature DLDGD was marked with red box. The highly conserved RNA recognition motif (RRM) and RNA-dependent RNA polymeras (RdRP) domains were indicated respectively in red and green lines. At, *Arabidopsis thaliana*; Nt, *Nicotiana tobacum*; Ng, *Nicotiana glutinosa*; Nb, *Nicotiana benthamiana*; Sl, *Solanum lycopersicum*; St, *Solanum tuberosum*. **(C)** Phylogenetic analyses of CaRDR1 and its homologs using MEGA5 software based on the neighbor joining method. CaRDR1 from pepper was indicated in red boxes.

Through BLAST analysis in Pepper Genome Platform (http://peppergenome.snu.ac.kr/), we identified a putative *RDR* homolog named as *CaRDR1* (Capan11g001709). *CaRDR1* was cloned using cDNA extracted from pepper leaves of line P79. The full-length *CaRDR1* cDNA consists of 3,351 bp and encodes 1116 amino acids, in which molecular mass of 127.4 kDa and isoelectric point of 8.51 were detected. The full-length of *CaRDR1* genomic sequence contains 5,217 bp, including four exons and three introns (Figure 1A). The sequence alignment of the amino acid residues of CaRDR1 compared with other members of the RDR1 family was performed using ClustalW in the MEGA5 software package (Tamura, 2011). CaRDR1 in pepper was 60.69, 86.56, 86.12, 87.99, and 88.90% identical to the RDR1s from Arabidopsis, tabacco, *Nicotiana glutinosa*, tomato and potato, respectively. The RNA-dependent RNA polymerase catalytic domain of CaRDR1 was located from His (370 aa position) to Val (892 aa position) and the RNA recognition motif (RRM) started from Ile (5 aa position) to Ile (62 aa position; Figure 1B). Furthermore, the RDR1s from various species share a signature motif DLDGD. In addition, CaRDR1 also contained the conserved components which are typical structures in RDR1 family (Wassenegger and Krczal, 2006; Bologna and Voinnet, 2014; Figure 1B).

In order to understand the evolutionary relationship between CaRDR1 and other RDRs homologs from various species, we performed phylogenetic analysis using MEGA5 software (Figure 1C). The results revealed that RDRs could be classified into four main groups. RDR1s from pepper, tomato, potato and tobacco, which belonged to solanaceae family, were fall into the same clade, suggesting that they might have similar functions. These observations revealed that *CaRDR1* was a *RDR1* homolog in pepper.

### Expression pattern of *CaRDR1* gene

**Figure 2:**
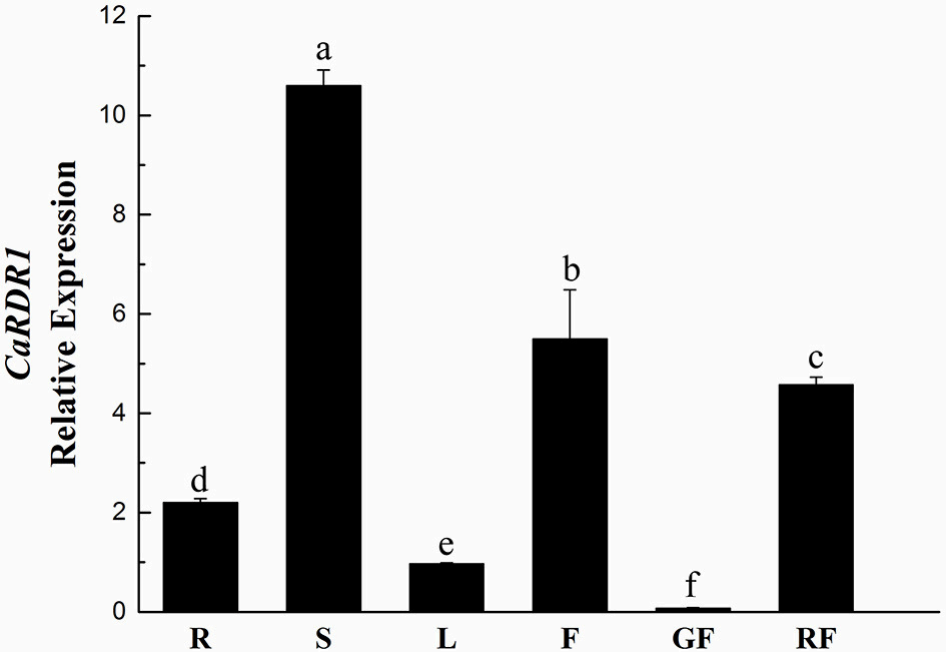
qRT-PCR analyses relative expression of *CaRDR1* in different tissues of pepper. Three biological replicates were performed for this experiment and the pepper *Ubi3* gene was used as the reference gene. Error bars indicate the standard errors. Letters indicate the significant differences (*P* < 0.05) between samples. R, roots; S, stems; L, leaves; F, flowers; GF, green fruits; GF, red fruits.

To further elucidate the function of *CaRDR1*, its expression pattern analysis was performed in roots, stems, leaves, flowers, green fruits, and red fruits of pepper P79 using qRT-PCR. As shown in Figure 2, *CaRDR1* transcript was expressed in all tissues examined, which was consistent with the vital roles of *RDR1* in plants. While the highest expression of *CaRDR1* was detected in the stems. Similar to *NbRDR1m* of *N. benthamiana* (Yang et al., 2004), transcript levels of *CaRDR1* in stems, roots and flowers were higher than that in leaves (Figure 2). It is to be noted that *AtRDR1* of Arabidopsis is mainly expressed in tissues with mature vascular system (Xu et al., 2013). These data indicated that *CaRDR1* might have an important role in limiting pathogen spread.

### *CaRDR1* was induced by phytohormones treatment and TMV infection

**Figure 3:**
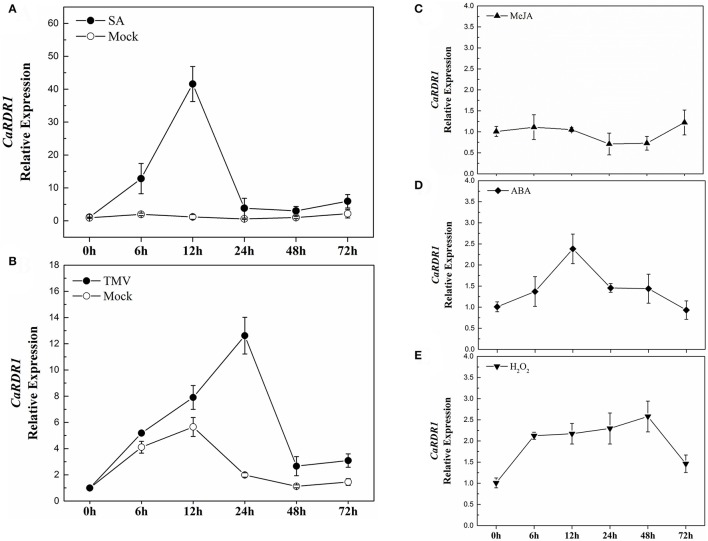
qRT-PCR analyses of *CaRDR1* expression as influenced by exogenous phytohormones and TMV inoculation in P79. **(A)** Effect of 2 mM SA on the expression of *CaRDR1* in pepper leaves. **(B)** Effect of TMV on the expression of *CaRDR1* in pepper leaves. **(C)** Effect of 100 μM MeJA on the expression of *CaRDR1* in pepper leaves. **(D)** Effect of 100 μM ABA on the expression of *CaRDR1* in pepper leaves. **(E)** Effect of 10 mM H_2_O_2_ on the expression of *CaRDR1* in pepper leaves. The pepper *Ubi3* was used as the reference gene, and three biological replicates were performed for these experiments. Error bars indicate the standard errors.

Since RDRs have been shown as a major constituents for the siRNAs production involved in plant response to stresses (Xie et al., 2001; Hannon, 2002; Yu et al., 2003; Baulcombe, 2004; Ding and Lu, 2011), we analyzed the response of *CaRDR1* gene to exogenous SA, MeJA, ABA, and H_2_O_2_ in both P79 and P54, which had different response to TMV infect (Figures S1A,B). The results showed that *CaRDR1* was induced by SA treatment in both P79 and P54 plants. The response of *CaRDR1* to SA increased then reaching the maximum induction at 12 h with a 42-fold increase and then declined in P79 (Figure 3A). Surprisingly, the expression patterns of *CaRDR1* were almost similar in P54 plants, where the transcript levels of *CaRDR1* peaked at 12 h with a 25-fold increase (Figure S1C). After ABA treatment, *CaRDR1* relative expression reached a maximum induction of 2.4-fold at 12 h (Figure 3D). The response of *CaRDR1* to H_2_O_2_ increased gradually, reached a peak at 48 h of 2.5-fold (Figure 3E), however, it was not induced by MeJA (Figure 3C). To our surprise, the transcripts of *CaRDR1* did not significantly response to MeJA, ABA, and H_2_O_2_ in P54 (data not show).

Furthermore, to examine the response of *CaRDR1* to TMV, at the 8-week-old, pepper leaves were inoculated with TMV-U1 train using mechanical inoculation technique, subsequently, the transcript level of *CaRDR1* was analyzed. Leaves inoculated with phosphate buffer were used as controls. As shown in Figure 3B, *CaRDR1* was up-regulated upon TMV infection, and the expression level of *CaRDR1* showed a 12-fold-increase after TMV inoculation for 24 h in P79, the highest transcript level was detected at 24 h when the transcript level of *CaRDR1* was induced to 9-fold in P54 (Figure S1D). These results indicated that *CaRDR1* might be involved in SA-modulated plant virus defense and participated in pepper interaction with TMV.

### Silencing of *CaRDR1* reduced the TMV resistance of pepper

**Figure 4:**
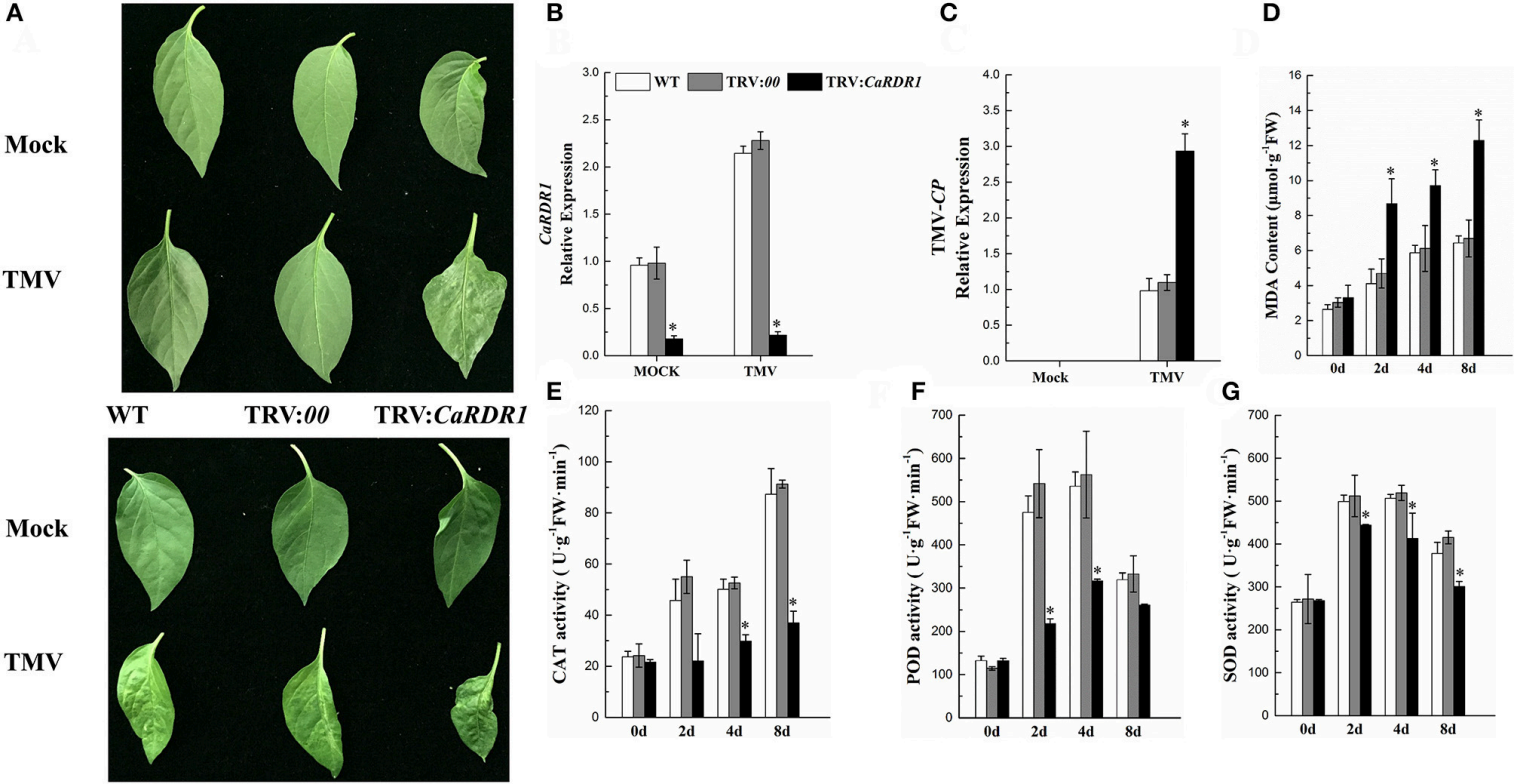
Silencing of *CaRDR1* attenuated the TMV resistance of pepper. **(A)** Phenotype of upper un-inoculated leaves from WT, empty vector (TRV:*00*), and *CaRDR1*-silenced (TRV:*CaRDR1*) plants, P79 (upper) and P54 (down) at 15 dpi with TMV. **(B,C)** qRT-PCR was used to determine the relative level of *CaRDR1* **(B)** and TMV*-CP* **(C)** transcript of WT, empty vector (TRV: *00*), and *CaRDR1*-silenced (TRV: *CaRDR1*) plants at 7 days post-inoculation (dpi). **(D)** The malondialdehyde (MDA) content. **(E)** catalase (CAT) activities **(F)** peroxidase (POD) activities **(G)** superoxide dismutase (SOD) activities. Error bars indicate the standard errors. Asterisks indicate the significant differences (*P* < 0.05) between WT, TRV:*00* and TRV: *CaRDR1* lines.

To determine the *CaRDR1* loss-of-function phenotype in pepper TMV defense, VIGS was performed to generate *CaRDR1*-silenced plants by using recombinant tobacco rattle virus (TRV) construct (Liu et al., 2002b) containing the specific 383 bp cDNA sequence. And the empty vector (EV) was also injected to pepper, that acted as the Control. qRT-PCR analysis revealed that the transcript level of the *CaRDR1* was reduced by 80% in the silenced plants, suggesting that *CaRDR1* was effectively knocked down by VIGS (Figure 4B, Figure S2A). The WT and TRV:*00* plants of P79 showed no symptom, but the upper un-inoculated leaves of *CaRDR1*-silenced plants exhibited chlorisis and mosaic 15 days post TMV inoculated (Figure 4A). The upper un-inoculated leaves of WT and TRV:*00* plants showed mosaic of P54, TRV:*CaRDR1* ones exhibited shrinking and mosaic (Figure 4B). Furthermore, the TMV-*CP* expression in leaves from the *CaRDR1*-silenced lines was increased by 3-fold as compared to that in the control plants after 7d of TMV incubation in P79, and 1.5-fold as compared to control in P54 (Figure 4C, Figure S2B). Moreover, some biochemical indices were examined in the CaRDR1 silenced plants, and we found that loss-of-function of *CaRDR1* could increase MDA production after TMV infection. For example, the MDA content was induced by TMV inoculation in both silenced lines and control plants, but the higher MDA accumulation was detected in *CaRDR1* silenced plants at all time points tested after TMV infection (Figure 4D, Figure S2C). These results suggested that plasma membrane damage was more serious in *CaRDR1*-silenced plants in both resistant and susceptible plants. Furthermore, after TMV infection, the SOD and POD activities were initially elevated and then decreased at 8 dpi in both silenced and control plants, but the activity of CAT was increased gradually. However, the activities of these three antioxidant enzymes in *CaRDR1*-silenced plants were significantly lower than that of control plants, indicating that knockdown of *CaRDR1* inhibited antioxidant enzyme activity under TMV inoculation (Figures 4E–G, Figures S2D–F). The patterns of biochemical indices were similar in P79 and P54, but the MDA accumulation was higher in P54 and *CaRDR1*-silenced plants than that of P79 plants at all time points tested after TMV infection (Figures S2C–F). The activities of these three antioxidant enzymes were highest in P79 control plants after TMV infection. Since SOD, POD, and CAT are directly involved in scavenging reactive oxygen species (ROS) (Chen et al., 1993; Montalbini et al., 1995; Agnieszka et al., 2009), *CaRDR1* modulated changes in their activities might indicate a positive role of *CaRDR1* in TMV defense of pepper.

### Overexpression of *CaRDR1* protected *N. benthamiana* from TMV-induced damage

**Figure 5:**
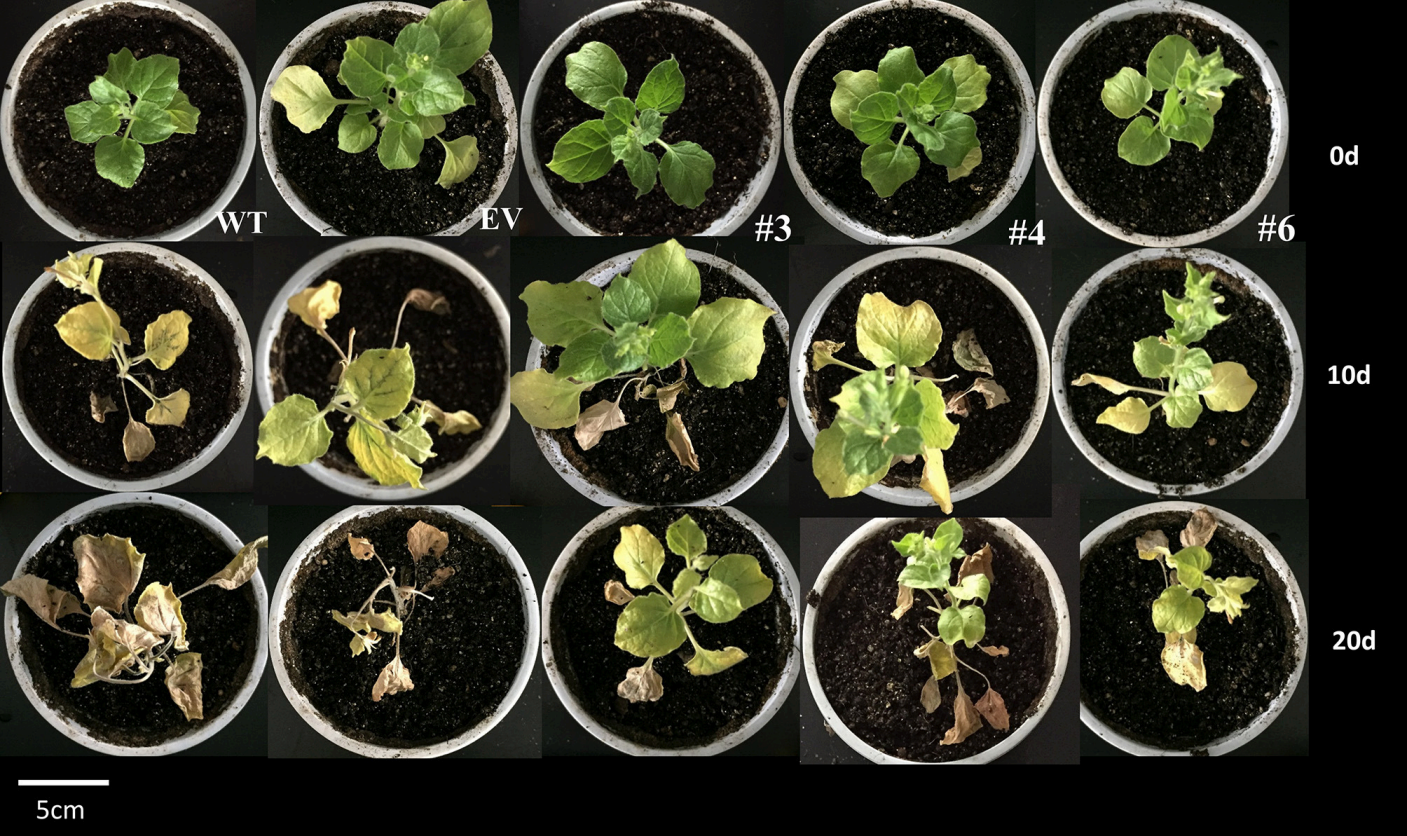
Phenotype analysis of wild type, empty vector and *CaRDR1*-overexpressed (OE-3, OE-4, and OE-6) plants at 0, 10, and 20 dpi with TMV.

**Figure 6:**
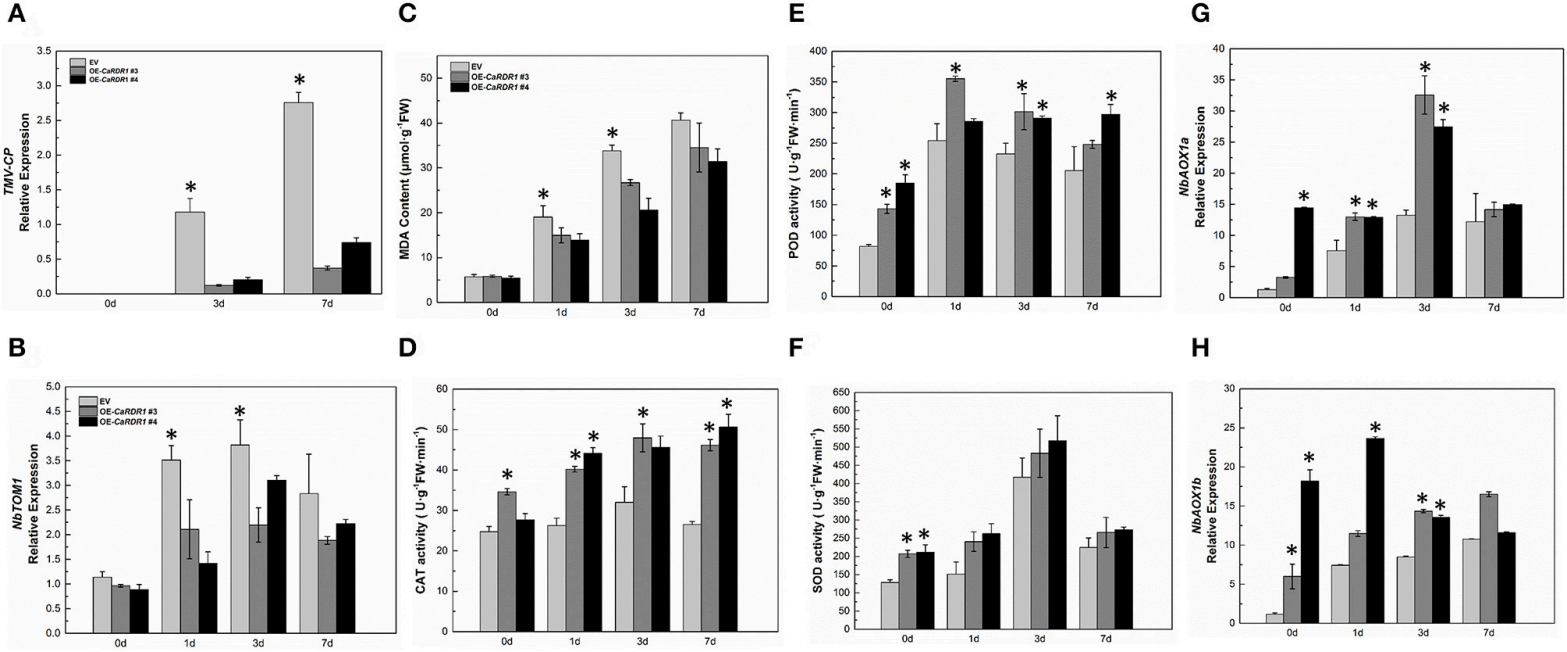
Overexpression *CaRDR1* enhanced the TMV resistance of *N. benthamiana*. **(A)** qRT-PCR was used to determine the relative level of TMV–*CP* of empty vector and *CaRDR1*-OE plants at 0, 3, and 7 dpi. **(B)** qRT-PCR was used to determine the relative level of *NbTOM* in upper un-inoculated leaves of empty vector and *CaRDR1*-OE plants at 0, 1, 3, and 7 dpi. **(C)** The MDA content **(D–F)** CAT **(D)**, SOD **(E)** and POD **(F)** activities measurement in leaves of empty vector and *CaRDR1*-OE plants at 0, 1, 3, and 7 dpi. **(G,H)** qRT-PCR was used to determine the relative level of *NbAOX1a* **(G)** and *NbAOX1b* **(H)** transcript in empty vector and *CaRDR1*-OE plants at 0, 1, 3, and 7 dpi. Three biological replicates were performed for these experiments. The *N. benthamiana NbEF1*α gene was used as the reference gene. Error bars indicate the standard errors. Asterisks indicate the significant differences (*P* < 0.05) between EV and *CaRDR1*-OE lines.

As silencing of *CaRDR1* reduced TMV defense of pepper, we hypothesized that overexpression of *CaRDR1* might enhance TMV resistance. Therefore, we generated individual *CaRDR1* overexpressing (*CaRDR1*-OE) transgenic *N. benthamiana* lines and EV line. We used real-time RT-PCR analysis to identify lines that expressed the transgene at high levels (Figure S3). It was observed that developing leaves from both *CaRDR1*-OE (OE-3, OE-4, OE-6) and EV plants became chlorotic and shrinking 10 days post-inoculation (dpi). Meanwhile, EV control plants displayed stem and leaf necrosis, however, such symptom was not observed on plants transformed with *CaRDR1*. By 20 dpi, EV plants were dead or near death, whereas *CaRDR1*-OE plants showed continued growth with yellowing and mosaic symptoms in young leaves (Figure 5). qRT-PCR was used to analyze the TMV-*CP* expression in leaves from the *CaRDR1*-OE lines. At 3 dpi, the transgenic lines expressed an increased transcript level of *CaRDR1* but a decreased transcript level of *CP* RNA compared with that of EV line (Figure 6A). By 7 dpi, accumulation of TMV-*CP* transcript was remarkably increased both in the transgenic and EV lines, but EV line showed a 3.5-fold increased transcript of TMV-*CP* compared to that of *CaRDR1*-OE lines (Figure 6A). In addition, *NbTOM1*, which is required for efficient multiplication of Tobamoviruses, was down-regulated in *CaRDR1*-OE plants as compared with EV plants (Figure 6B). Likewise, from 1 to 3 dpi, a decreased MDA content was detected in leaves of *CaRDR1*-OE lines compared to EV plants (Figure 6C). As plants possess antioxidants that can scavenge ROS to protect cells from oxidative damage, the activities of SOD, POD and CAT were investigated in both *CaRDR1*-OE and EV plants after TMV inoculation. The activities of CAT and POD in the *CaRDR1*-OE lines kept higher than in the EV plants, but the TMV treatment resulted in non-significant increase of the activities of CAT in both *CaRDR1*-OE and EV ones (Figures 6D,E). The *CaRDR1*-OE lines exhibited high SOD activity in un-treatment plants, but non-significant difference with EV lines under TMV infection. qPCR analysis showed that the TMV infection increased the relative mRNA abundance of *NbAOX1a* in *CaRDR1*-OE of 2.5-fold compared to EV plants at 3 dpi. *NbAOX1b* did not responded to TMV (Figures 6G,H).

**Figure 7:**
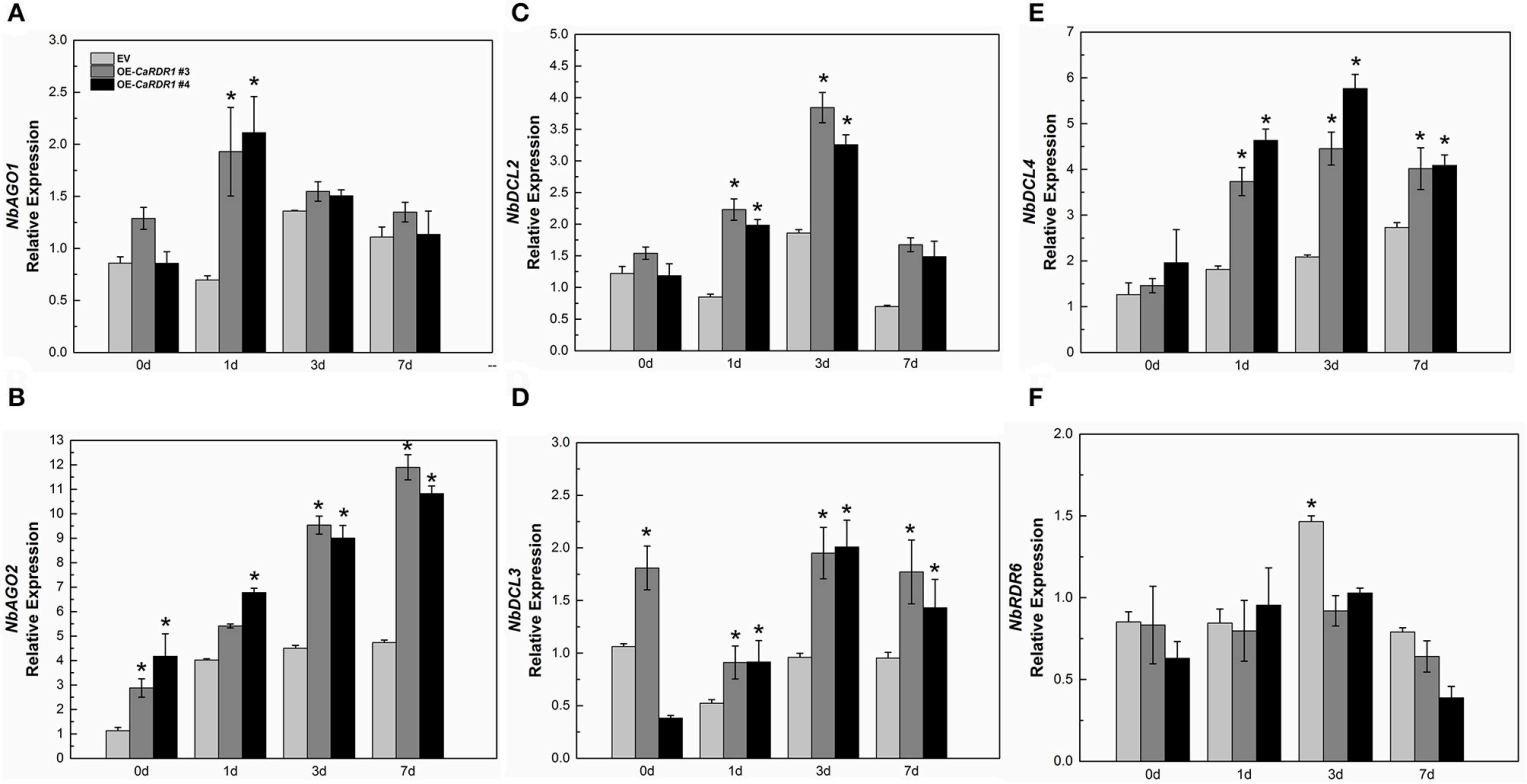
RNA silencing related genes expression in EV and *CaRDR1*-OE plants after TMV inoculation **(A–F)**. **(A)** *NbAGO1* **(B)** *NbAGO2* **(C)** *NbDCL2* **(D)** *NbDCL3* **(E)** *NbDCL4* **(F)** *NbRDR6*. The leaf samples were obtained from empty vector and *CaRDR1*-OE plants at 0, 1, 3, and 7 dpi. Three biological replicates were performed for these experiments. The *N. benthamiana NbEF1*α gene was used as the reference gene. Error bars indicate the standard errors. Asterisks indicate the significant differences (*P* < 0.05) between EV and *CaRDR1*-OE lines.

*NbAGO1, NbAGO2, NbDCL2, NbDCL3, NbDCL4, NbRDR6*, and *NbRDR1* participate in RNA silencing-mediated virus defense conjointly (Nakasugi et al., 2013). Since our results reveal that *CaRDR1* is positively correlated with TMV resistance, we then intended to clarify whether *CaRDR1* affects the expression profiles of RNA silencing genes. The expression patterns of *NbAGO1, NbAGO2, NbDCL2, NbDCL3, NbDCL4*, and *NbRDR6* were monitored in *CaRDR1*-OE and EV plants following TMV inoculation. The overexpression of *CaRDR1* showed up-regulated of *NbAGO2*. TMV inoculation induced up-regulation of *NbAGO1, NbAGO2, NbDCL2*, and *NbDCL4*. The expression of *NbAGO1* was two times higher in *CaRDR1*-OE plants than that in EV plants at 1 dpi (Figure 7A). TMV inoculation gradually increased transcript of *NbAGO2* over time, which remained consistently higher in *CaRDR*1-OE plants than that in EV plants (Figure 7B). The magnitude of *NbDCL2/4* were also higher in *CaRDR1*-OE plants during TMV infection. The highest transcript levels were detected at 3 dpi, when the transcript of *NbDCL2/4* were induced to 2- and 3-fold, respectively in *CaRDR1*-OE plants compared to EV (Figures 7C,E). In contrast, expression of *NbRDR6* was downregulated in *CaRDR*1-OE plants, particularly at 3 dpi (Figure 7F). The data presented here indicate that *CaRDR1* might play an important role in regulating these RNA silencing related genes upon TMV inoculation. Taken together, these results suggested that *CaRDR1* functions positively in TMV resistance by up-regulating *AGOs* and *DCLs* in *N. benthamiana*.

## Discussion

In the present study, a *CaRDR1* gene was cloned from pepper. The expression of *CaRDR1* was induced by SA, ABA, H_2_O_2_, and TMV. Further, down-regulated the transcripts of *CaRDR1* through VIGS caused severe symptom and more virus RNA accumulation in pepper. Ectopic expression *CaRDR1* in *N. benthamiana* suppressed the lethal damage of TMV, in addition enhanced the expression of RNA silencing-related genes. The data suggested that *CaRDR1* might act as a positive regulator in the interaction of pepper and TMV.

RNA silencing functions as a major natural antiviral defense mechanism in plants, but there are little studies of this process in pepper. RDRs synthesize dsRNA intermediates and play an important role in the initiation and amplification of RNA silencing (Baulcombe, 2004; Ding and Lu, 2011). There are six RDRs in *Arabidopsis thaliana*, AtRDR1, AtRDR2, AtRDR3a, AtRDR3b, AtRDR3c, and AtRDR6 (Wassenegger and Krczal, 2006). Several recent researches indicate that plant RDR1 is involved in antiviral defense (Diaz-Pendon et al., 2007; Qi et al., 2009; Qu, 2010). *RDR1* orthologs have been identified in many species, such as Arabidopsis (*AtRDR1*), tomato (*SlRDR1*), *Nicotiana* spp. (*NtRDR1, NbRDR1*, and *NgRDR1*), potato (*StRDR1*), rice (*OsRDR1*), and maize (*ZmRDR1*; Schiebel et al., 1998; Xie et al., 2001; Yu et al., 2003; Yang et al., 2004; Wassenegger and Krczal, 2006; Liu et al., 2009; Hunter et al., 2013). Nonetheless, the functions of *RDR1* still remain disputed in different hosts and viruses. In this study, a *CaRDR1* gene was identified from pepper. Similar to other RDR1s, CaRDR1 also has a signature catalytic motif DLDGD (Figure 1B), which is likely to form the nucleotidyl transferase active site partially via a coordinated divalent cation (Iyer et al., 2003; Wassenegger and Krczal, 2006). Phylogenetic analysis indicated that RDR1 proteins in the solanaceae family fall into the same clade (Figure 1C). Investigated its expression pattern in different plant parts, it was found that *CaRDR1* was predominantly expressed in pepper stem (Figure 2). As the previous research, *NbRdRP1m* in *N. benthamiana* expressed higher in stem than leaf, moreover, *AtRDR1* was expressed in tissues with mature vascular system in phloem (Yang et al., 2004; Xu et al., 2013). The vascular system of plants has been shown to play an important role in virus spread (Lough and Lucas, 2006; Petricka et al., 2012). Considering the positive role of *CaRDR1* in defense against TMV, we proposed that the high *CaRDR1* expression in the stem might limit pathogen spread, leading to an enhancement in pepper resistance against virus.

Plant hormones play a vital role in plant immunity. In pathogens defense, plants produce a complicated mixture of SA, JA, and ABA to modulate plant defense response to invading pathogens (Kunkel and Brooks, 2002; Chen et al., 2013; He et al., 2017). *CaRDR1* was response to multiple plant hormones in this study (Figure 3), especially induced by SA like other *RDR1* orthologs (Yang et al., 2004; Hunter et al., 2013; Liao et al., 2013). Interestingly, the expression of *CaRDR1* could not be induced by MeJA treatment. Nonetheless, *NgRDR1* in *Nicotiana glutinosa* was up-regulated by MeJA (Liu et al., 2009). The interactions between SA and JA signaling are complex, the primary mode of interaction between them appears to be mutual antagonism (Kunkel and Brooks, 2002). It is suggested that *CaRDR1* involved in pepper TMV resistance cooperated with SA rather than with JA. *CaRDR1* also could be induced by ABA and H_2_O_2_, which suggests that *CaRDR1* may interact with SA, H_2_O_2_, and ABA signal pathways. The different expression patterns of *RDRs* upon treatments with different signaling molecules indicate the complexity of the RDR family in higher plants, and also suggest that different *RDRs* may participate in different signal pathways and have different functions.

*CaRDR1* was induced by TMV infection in both TMV resistant and susceptible pepper genotypes. While knockdown of *CaRDR1* in leaves by VIGS increased the transcript level of TMV-*CP* and reduced the resistance to TMV of pepper (Figure 4). This is consistent with previous reports that *NtRDRP1* antisense transgenic tobacco accumulated significantly higher levels of viral and displayed more severe disease symptoms (Xie et al., 2001). The *AtRDR1* knockout mutant accumulated higher persistent viral RNAs levels after infected by a tobamovirus and a tobravirus in Arabidopsis (Yu et al., 2003). However, suppression the transcripts of *StRDR1* in potato did not increase the susceptibility of potato to PVX, PVY, and TMV (Hunter et al., 2016). MDA is one of the important lipid peroxidation products involved in the plant defense singling under many stresses (Gechev et al., 2002; War et al., 2012). While our data displayed that down-regulation of *CaRDR1* led to an increasing of MDA content, and overexpression of *CaRDR1* led to MDA content decreased (Figures 4D, 6C). Increased MDA content are often associated with oxidative stress (Gechev et al., 2002; Madhusudhan et al., 2009). It suggested that down-regulation of *CaRDR1* subsequent damage to the plasma membrane. Attenuated antioxidant enzymes activities were obtained in *CaRDR1*-silencied plants compared to the control ones under TMV infection (Figures 4E–G). It has been reported that TMV infection could induce POD activity but suppress CAT activity in tobacco, bell pepper and tomato plants (Madhusudhan et al., 2009). POD activity increased in *RDR1* silenced tobacco lines 3 days after inoculation with PVY (Rakhshandehroo et al., 2012). ROS perform multiple roles during plant defense responses to microbial attack by acting directly in the initial defense (Klessig et al., 2000). On the other hand, ROS accumulating overly shown to be toxic. Antioxidant enzymes regulate the content of ROS depends on its role in virus defense. Therefore, the activities of antioxidant enzymes may be influenced by the period of measurement. The VIGS-silencing experiment implied that the role of *RDR1* in the defense response was different among crops. There are some disparities among the *RDR* gene family in different plants species. The redundant and overlap of *RDRs* functions might be one explanation for these results.

TMV-U1 strain causes the death of *N. benthamiana* plants. *CaRDR1* overexpression showed moderate disease symptom and delayed the lethality of TMV-U1 on *N. benthamiana* plants (Figure 5). Furthermore, *CaRDR1*-OE transgenic *N. benthamiana* showed less TMV-*CP* transcripts than EV ones (Figure 6A). These results agreed with previous studies that *MtRDR1* overexpression provided protection against TMV infection, which eventually kept tissue adjacent to the apical meristem free from TMV particles (Yang et al., 2004; Lee et al., 2016). TOM1 interacts with the helicase domain of TMV replicase to form the replication complex, and supports tobamovirus multiplication on an early stage of infection with TOM3 (Hagiwara et al., 2003; Asano et al., 2005). In addition, the expression of *NbTOM1* which required for efficient multiplication of Tobamoviruses decreased in *CaRDR1*-OE lines (Figure 6B), suggesting that *CaRDR1* not only limited pathogen spread, but also suppressed TMV replication. *CaRDR1* overexpression alleviated the virus damage on the plasma membrane by less MDA accumulation (Figure 6C). The activity of CAT was higher in *CaRDR1*-OE lines than in EV plants (Figure 6D). Curiously, we observed that *CaRDR1* transgenic plants compared with WT plants had greater POD and SOD activities without the TMV infection (Figures 6E,F). The CaMV35S promoter was used in *CaRDR1* transgenic, it suggested that the constitutive overexpression of *CaRDR1* influenced the action mechanism of the antioxidant enzymes. Plant mitochondria possess a second terminal oxidase, termed the alternative oxidase (AOX). AOX is a terminal oxidase of the plant mitochondrial electron transport chain, which has an important function in plant defense against TMV (Chivasa et al., 1997; Fu et al., 2010; Lee et al., 2011; Liao et al., 2012). The expression of *NbAOX1a* and *NbAOX1b* were distinctly higher in *CaRDR1*-overexpressing lines, and TMV up-regulated the transcripts of *NbAOX1a* indicating a potential involvement of *CaRDR1* in the AOX-mediated defense response to TMV (Figures 6G,H). There is crosstalk between the functional RDR1-mediated signal pathway and the AOX pathway. ROS might be involved in the crosstalk between RDR1 and AOX. Overexpression of *CaRDR1* may altered *AOX* gene expression by influencing the action mechanism of antioxidant enzymes and the content of ROS. It is suggested *NbAOX1a* work as an assist for antioxidant systems after TMV challenge, restricting the accumulation of ROS, which might injure the plant membrane system.

The RNA silencing pathways components are encoded by gene families of the *DCLs, AGOs*, and *RDRs*. They accomplish targeted RNA degradation, translational repression and heterochromatin modification (Eamens et al., 2008). In this study, expression levels of *NbAGO1, NbAGO2, NbDCL2, NbDCL3*, and *NbDCL4* were relatively higher in transgenic *N. benthamiana* (Figure 7). *NbAGO1* and *NbAGO2* might participate in the interaction between *N. benthamiana* and TMV with *RDR1*. This agreed with a previous report that southern rice black-streaked dwarf virus (SRBSDV) infection significantly increased the expression of *OsAGO1d, OsAGO2, OsRDR1*, and *OsRDR6* (Xu and Zhou, 2017). RDR mediated the amplification process of RNA silencing, which is likely to be essential in virus defense to ensure the viral RNAs silencing keep pace with the replication of viral RNA (Baulcombe, 2004). Previous research showed that dsRNA was synthesized by host RDR1 or RDR6, recognized by DCL4 or DCL2, and processed into the secondary viral siRNAs to direct more potent antiviral silencing (Wang et al., 2010). It suggested the up-regulated *NbDCL2* and *NbDCL4* might be consociation with the overexpression *CaRDR1* to suppress the virus accumulation in *N. benthamiana*. Surprisingly, the expression of *NbRDR6* was reduced in *CaRDR1*-OE plants. *NtRDR1* might have a dual role, on one hand, contributing to SA mediated antiviral defense, on the other hand, suppressing the RDR6-mediated antiviral RNA silencing (Ying et al., 2010). *AtRDR6* was involved in cucumber mosaic virus (CMV) defense (Dalmay et al., 2000; Mourrain et al., 2000). *NbRDR6* had been discovered having effects on limiting the virus systemic spread during the Potato potexvirus X (PVX) infection (Schwach et al., 2005). In this study, *CaRDR1* might have a combination with *NbAGO2, NbDCL2/3/4* and complementary effect with *NbRDR6*. Owing to the apparent function of *CaRDR1* within different regulatory networks, it is difficult to define the detailed and specific individual pathway in TMV response. Therefore, further study is essential to gain a more thorough knowledge of roles of *CaRDR1* in these pathways and to elucidate the regulatory mechanisms more precisely.

## Conclusions

*CaRDR1* exhibited a high degree of identity with other RDR1s of *Solanaceae*. The transcripts of *CaRDR1* was induced by TMV and SA. *CaRDR1* also responded to H_2_O_2_ and ABA. When *CaRDR1* was silenced in pepper plants, the silenced plants showed increased TMV-*CP* transcript and MDA content but decreased antioxidant enzymes activities. In contrast, *CaRDR1*-OE plants showed mild symptom, decreased TMV-*CP* transcripts and elevated expression of RNA silencing related genes, including *NbAGO2, NbDCL2*, and *NbDCL4*. *CaRDR1* was likely to limit pathogen spread and suppress TMV replication to protect plant from TMV attack.

## Author contributions

YL and LQ designed the study. LQ, NM, and YangZ performed the experiments. LQ, TM, and GZ analyzed the data, LQ wrote original manuscript, YL and YanZ revised the manuscript, and YL gave the final approval of the version.

### Conflict of interest statement

The authors declare that the research was conducted in the absence of any commercial or financial relationships that could be construed as a potential conflict of interest.

## Supplementary material

Table S1 The sequences of primers used in this study.

Table S2 The sequence information of the proteins used for phylogenetic tree construction.

Figure S1 qRT-PCR analyses of *CaRDR1* transcript as influenced by exogenous SA treatment and TMV inoculation in P79 and P54 pepper genotypes. **(A)** Disease incidence (%) of TMV in P79 and P54 **(B)** Disease index of TMV in P79 and P54 **(C)** Effect of SA on the expression of *CaRDR1* in pepper leaves. **(D)** Effect of TMV on the expression of *CaRDR1* in pepper leaves. The pepper *Ubi3* was used as the reference gene, and three biological replicates were performed for these experiments. Err bars indicate the standard errors. Asterisks indicate the significant differences (*P* < 0.05) between P79 and P54.

Figure S2 Silencing of CaRDR1 attenuated the TMV resistance of pepper in P79 and P54 pepper genotypes. **(A,B)** qRT-PCR was used to determine the relative level of *CaRDR1* in P54 **(A)** and TMV −*CP* **(B)** transcript in un-inoculated leaves of empty vector (TRV: *00*) and CaRDR1-silenced (TRV: *CaRDR1*) plants at 7 days post-inoculation (dpi). **(C)** The MDA content measurement in inoculated leaves of empty vector (TRV: *00*) and CaRDR1 -silenced (TRV: *CaRDR1*) plants. **(D,F)** POD **(D)**, CAT **(E)**, and SOD **(F)** activities measurement in in inoculated leaves of empty vector (TRV: *00*) and CaRDR1 silencing (TRV: *CaRDR1*) plants. Three biological replicates were performed for these experiments. Error bars indicate the standard errors. Asterisks indicate the significant differences (*P* < 0.05) between TRV: *00* and TRV: *CaRDR1* lines.

Figure S3 Molecular characterization of transgenic plants. **(A)** PCR confirmation of transgenic *N. benthamiana* plants. Lanes: EV PBI121 vector; M molecular size marker; V CaRDR1-PBI121 vector; WT, untransformed wild type; OE-2,3,4,6,7,9 independent transgenic lines. **(B)** qRT-PCR analysis of *CaRDR1* mRNA levels from leaves of 4-week-old transgenic *N. benthamiana*. Three biological replicates were performed for this experiment and the *N. benthamiana NbEF1*α gene was used as the reference gene Err bars indicate the standard errors. Asterisks indicate the significant differences (P < 0.05) between WT, EV and *CaRDR1*-OE lines.

Presentation S1 Disease evaluation in pepper plants.

## Abbreviations

ABA: abscisic acid
AGO: argonaute
AOX: alternative oxidase
CAT: catalase
CMV: cucumber mosaic virus
DCL: dicer-like
EV: empty vector
LSD: least significant difference
MDA: malondialdehyde
POD: peroxidase
qRT-PCR: quantitative real-time PCR
PVX: potato potexvirus X
RDR: RNA-dependent RNA polymerases
ROS: reactive oxygen species
RRM: RNA recognition motif
SA: salicylic acid
SOD: superoxide dismutase
SRBSDV: southern rice black-streaked dwarf virus
TBARS: thiobarbituric acid-reactive substances
TMV: tobacco mosaic virus
TMV-CP: tobacco mosaic virus-coat protein
TRV: tobacco rattle virus
VIGS: virus-induced gene silencing
viRNAs: virus derived small interfering RNAs
