# Supplementary material for: CaRDR1, an RNA-Dependent RNA Polymerase Plays a Positive Role in Pepper Resistance against TMV
Source: Front Plant Sci. 2017 Jun 28;8:1068. doi: 10.3389/fpls.2017.01068 (PMC5487767; doi:10.3389/fpls.2017.01068)
Supplement: Table S1 — The sequences of primers used in this study. [file Table1.DOCX]

**Supplementary Material**

**CaRDR1, an RNA-dependent RNA polymerase plays a positive role in pepper resistance against TMV**

**Lei Qin, Ning Mo, Yang Zhang, Tayeb Muhammad, Guiye Zhao, Yan Zhang, Yan Liang***

*** Correspondence:**

**Yan Liang**

[**liangyan@nwsuaf.edu.cn**](mailto:liangyan@nwsuaf.edu.cn)

| Primer number | Primer name | Primer sequence (5’to 3’)  Sequences underlined are digestion sites |
| --- | --- | --- |
| 1 | CaRDR1-F | gctctagaatgggtaagacaattcaggtttc |
| 2 | CaRDR1-R | tccccccgggttatttcaacactaatgtgcgactc |
| 3 | CaRDR1-qRTPCR-F | Ttatttggtgctcctcgggtcta  ttcggagaaagcaaaggtgaaac |
| 4 | CaRDR1-qRTPCR-R |  |
| 5 | CaRDR1-VIGS-F | cgcggatccaatgttctgggagtgatttggatgg |
| 6 | CaRDR1-VIGS-R | cggggtaccgtgataggttggcttgtctggctta |
| 7 | CaRDR1-OE-F | ctaacagaactcgccgtaaagac |
| 8 | CaRDR1-OE-R | cctcctactagccaaacagatga |
| 9 | TMV-CP-qRTPCR-F | tcttgtcatcagcgtgggc |
| 10 | TMV-CP-qRTPCR-R | aacagtgctgtgactagcgggt |
| 11 | CaUBI3-qRTPCR-F | tgtccatctgctctctgttg  caccccaagcacaataagac |
| 12 | CaUBI3-qRTPCR-R |  |
| 13 | NbAGO1-qRTPCR-F | attgctgcggtggttgcttct |
| 14 | NbAGO1-qRTPCR-R | gcggcttctgtccagttgctc |
| 15 | NbAGO2-qRTPCR-F | aaggagcatttagataaggattcag |
| 16 | NbAGO2-qRTPCR-R | ttcagcccgtaccatttcacata |
| 17 | NbDCL2-qRTPCR-F | ggaagtagcggctttgtcat |
| 18 | NbDCL2-qRTPCR-R | gcatcgtggaatctcaggtag |
| 19 | NbDCL3-qRTPCR-F | agtcaacagagcgtaaatccaag |
| 20 | NbDCL3-qRTPCR-R | aaaccgtagtcgggtatagtca |
| 21 | NbDCL4-qRTPCR-F | ttttatcccaacaacttctacg |
| 22 | NbDCL4-qRTPCR-R | tcttacgcaaccactgatgaca |
| 23 | NbRDR6-qRTPCR-F | gctgtagatgttcctggttta |
| 24 | NbRDR6-qRTPCR-R | cttactgggtgggatgagat |
| 25 | NbTOM-qRTPCR-F | caccaggctaggagtttacca |
| 26 | NbTOM-qRTPCR-R | tcttcccaatgaattccaca |
| 27 | NbAOX1a-qRTPCR-F | cttcttcaacgcctatt |
| 28 | NbAOX1a-qRTPCR-R | cagccctaacaaccaa |
| 29 | NbAOX1b-qRTPCR-F | gaatgataagcagcacg |
| 30 | NbAOX1b-qRTPCR-R | tgacggtccaataagc |
| 31 | NbEF1α-qRTPCR-F | tggtgtcctcaagcctggtat |
| 32 | NbEF1α-qRTPCR-R | acgcttgagatccttaaccgc |

**Supplementary Tables**
Table S1. The sequences of primers used in this study

| Gene Name | Accession Number |
| --- | --- |
| AtRDR1 | BAF00633 |
| NtRDR1 | CAA09697 |
| NgRDR1 | ABV24845 |
| NbRDR1 | AAS78669 |
| SlRDR1 | CAA71421 |
| StRDR1 | XP_015160221 |
| VvRDR1 | XP_002284914 |
| BrRDR1 | XP_009148868 |
| CmRDR1 | XP_008465250 |
| CsRDR1 | NP_001267692 |
| ZmRDR1 | XP_008644240 |
| HvRDR1 | ACH53360 |
| OsRDR1 | XP_015627028 |
| GmRDR1 | XP_003518619.1 |
| MtRDR1 | XP_013466603 |
| MdRDR1 | XP_008381760 |
| ObRDR1 | XP_006647856 |
| PtRDR1 | AJP06345 |
| AtRDR2 | NP_192851 |
| NtRDR2 | XP_016448058 |
| SlRDR2 | XP_004236120 |
| StRDR2 | XP_006345040.1 |
| NtRDR3 | XP_016511895 |
| SlRDR3 | XP_010322170 |
| StRDR3 | XP_006338861 |
| NtRDR5 | XP_016449580 |
| SlRDR5 | XP_010313737 |
| StRDR5 | XP_006353487 |
| AtRDR6 | NP_190519 |
| NtRDR6 | ADI52625 |
| SlRDR6 | NP_001266205 |
| StRDR6 | XP_006346784 |

Table S2. The sequence information of the proteins used for phylogenetic tree construction
