# Supplementary material for: CaRDR1, an RNA-Dependent RNA Polymerase Plays a Positive Role in Pepper Resistance against TMV
Source: Front Plant Sci. 2017 Jun 28;8:1068. doi: 10.3389/fpls.2017.01068 (PMC5487767; doi:10.3389/fpls.2017.01068)
Supplement: Figure S1 — qRT-PCR analyses of CaRDR1 transcript as influenced by exogenous SA treatment and TMV inoculation in P79 and P54 pepper genotypes. (A) Disease incidence (%) of TMV in P79 and P54 (B) Disease index of TMV in P79 and P54 (C) Effect of SA on the expression of CaRDR1 in pepper leaves. (D) Effect of TMV on the expression of CaRDR1 in pepper leaves. The pepper Ubi3 was used as the reference gene, and three biological replicates were performed for these experiments. Err bars indicate the standard errors. Asterisks indicate the significant differences (P < 0.05) between P79 and P54. [file Image1.PDF]

Supplementary Material

CaRDR1, an RNA-dependent RNA polymerase plays a positive role in pepper resistance against TMV

Lei Qin, Ning Mo, Yang Zhang, Tayeb Muhammad, Guiye Zhao, Yan Zhang, Yan Liang*

*** Correspondence:**

Yan Liang

liangyan@nwsuaf.edu.cn

Supplementary Figures

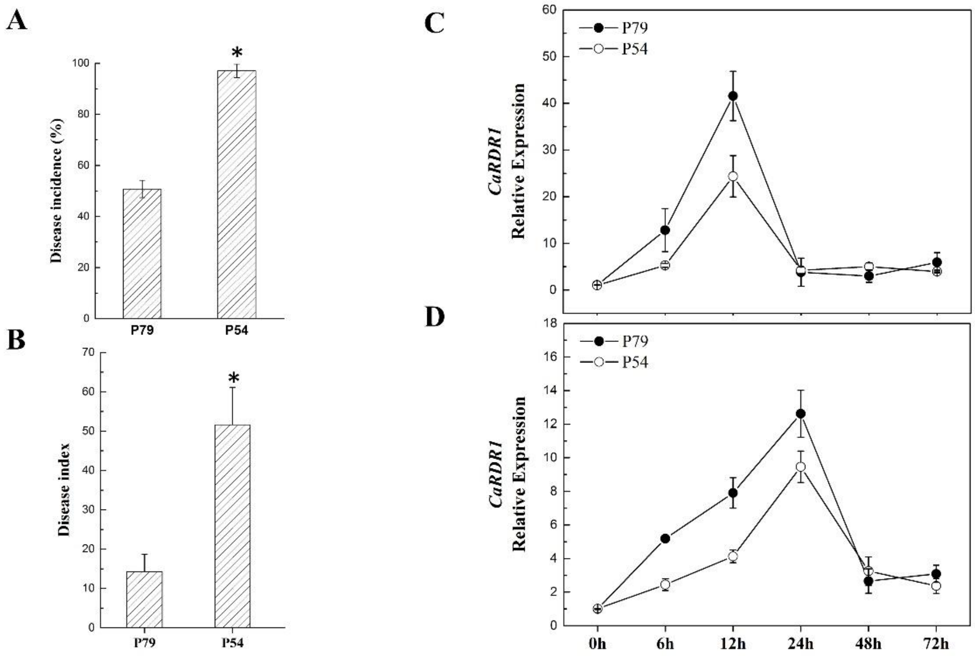

Figure S1. qRT-PCR analyses of *CaRDR1* transcript as influenced by exogenous SA treatment and TMV inoculation in P79 and P54 pepper genotypes. (A) Disease incidence (%) of TMV in P79 and P54 (B) Disease index of TMV in P79 and P54 (C) Effect of SA on the expression of *CaRDR1* in pepper leaves. (D) Effect of TMV on the expression of *CaRDR1* in pepper leaves. The pepper *Ubi3* was used as the reference gene, and three biological replicates were performed for these experiments. Err(A) Disease index of TMV in P79 and P54or bars indicate the standard errors.

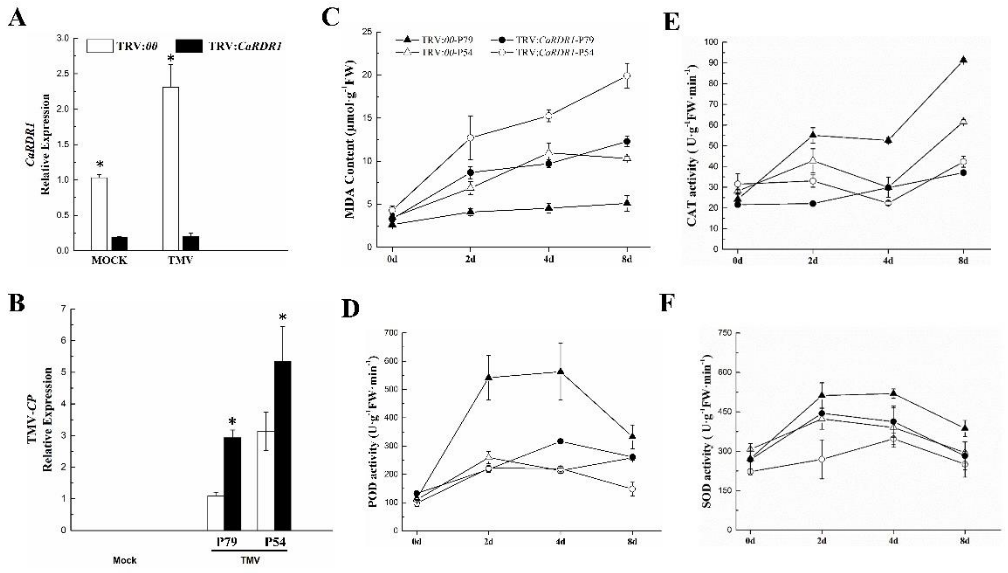

Figure S2. Silencing of *CaRDR1* attenuated the TMV resistance of pepper in P79 and P54 pepper genotypes. (A-B) qRT-PCR was used to determine the relative level of *CaRDR1* in P54 (A) and TMV -*CP* (B) transcript in un-inoculated leaves of empty vector (TRV: 00) and *CaRDR1*-silenced (TRV: *CaRDR1*) plants at 7 days post-inoculation (dpi). (C) The MDA content measurement in inoculated leaves of empty vector (TRV: *00)* and *CaRDR1* -silenced (TRV: *CaRDR1)* plants. (D-E) POD (D), CAT (E), and SOD (F) activities measurement in in inoculated leaves of empty vector (TRV: 00) and *CaRDR1* silencing (TRV: *CaRDR1)* plants. Three biological replicates were performed for these experiments. Error bars indicate the standard errors. Asterisks indicate the significant differences (P<0.05) between TRV:*00* and TRV: *CaRDR1* lines.

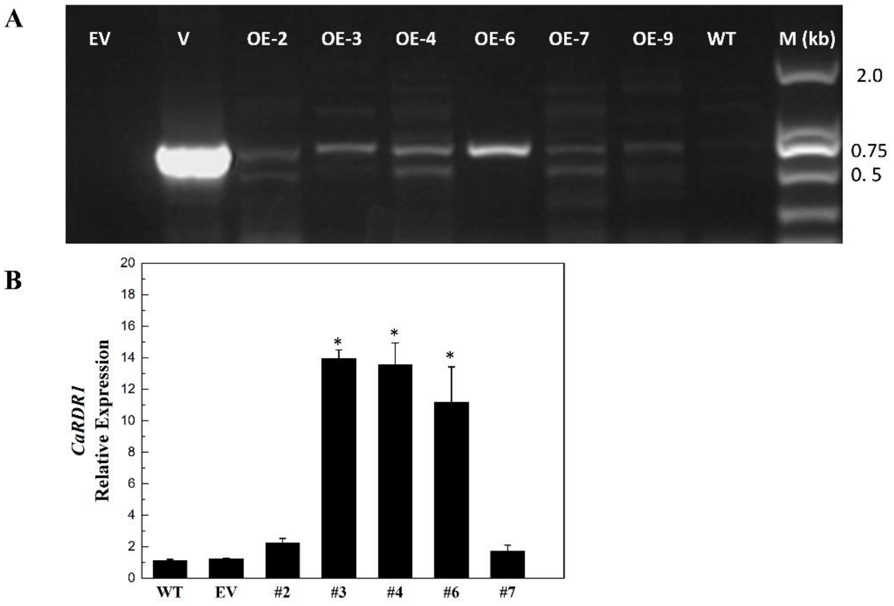

Figure S3. Molecular characterization of transgenic plants. (A) PCR confirmation of transgenic *N. benthamiana* plants. Lanes: EV PBI121 vector; M molecular size marker; V CaRDR1-PBI121 vector; WT, untransformed wild type; OE-2,3,4,6,7,9 independent transgenic lines. (B) qRT-PCR analysis of *CaRDR1* mRNA levels from leaves of 4-week-old transgenic *N. benthamiana*. Three biological replicates were performed for this experiment and the *N. benthamiana NbEF1α* gene was used as the reference gene.
