# Supplementary material for: CaRDR1, an RNA-Dependent RNA Polymerase Plays a Positive Role in Pepper Resistance against TMV
Source: Front Plant Sci. 2017 Jun 28;8:1068. doi: 10.3389/fpls.2017.01068 (PMC5487767; doi:10.3389/fpls.2017.01068)
Supplement: Presentation S1 — Disease evaluation in pepper plants. [file Presentation1.PDF]

Supplementary Material

CaRDR1, an RNA-dependent RNA polymerase plays a positive role in pepper resistance against TMV

Lei Qin, Ning Mo, Yang Zhang, Tayeb Muhammad, Guiye Zhao, Yan Zhang, Yan Liang*

*** Correspondence:**

Yan Liang

liangyan@nwsuaf.edu.cn

Supplementary Presentation

Disease evaluation in pepper plants

The disease incidence (%) and disease index were determined at 30 days post TMV inoculation (dpi) in pepper. The calculation of disease incidence (%) and disease index were as follows. Disease incidence = Number of plants with disease symptom/Number of all tested plants × 100%. Disease index = [Σ (Number of plants in a scale ×Corresponding scale value) / (Total number of plants × Highest scale value)] ×100.

Disease severity was evaluated using a rating scale of 0 to 9, in which, 0=No visible symptoms, 1=Local necrotic spots on the inoculated leaves, 3=Mild mosaic/mottling on few leaves of a plant, 5=Moderate mosaic/puckering/mottling on many leaves of a plant, 7=Severe mosaic/puckering/mottling and stunting, 9=Severe stunting and death in most leaves of infected plants.

Supplementary Reference

Li, X.X., and Zhang, B.X. (2006). Descriptors and Data Standard for Capsicum. Bei Jing: China Agriculture Press.
